# Using the knife to build the trust? The role of trust in the decision-making process of aesthetic surgeons and women patients/clients

**DOI:** 10.3389/fsoc.2024.1491948

**Published:** 2025-01-17

**Authors:** Michaela Honelová, Peta Cook, Lucie Vidovićová

**Affiliations:** ^1^Charles University, Faculty of Humanities, Department of Longevity Studies, Prague, Czechia; ^2^University of Tasmania, School of Social Sciences, Hobart, TAS, Australia

**Keywords:** aesthetic surgery, cosmetic surgery, medical encounter, aesthetic encounter, gender, trust, decision-making, Czech Republic

## Abstract

Trust is a fundamental element in decision-making processes. In medicine, trust also helps to build relationships between patients/clients and doctors (aesthetic surgeons) and will influence a woman’s decision to undergo aesthetic/cosmetic surgery. Patients/clients, as well as aesthetic surgeons, use different ways to build trust. Our analyses are based on fifteen qualitative interviews with aesthetic surgeons, fifteen qualitative interviews with women who have undergone or are planning to undergo aesthetic surgery procedure(s) and non-participatory observations at the clinic of aesthetic surgery in the Czech Republic. Based on our analysis, three levels of trust were identified: macro level: trust in medicine as a social institution; meso level: *a priori* trust to the aesthetic surgeon; and micro level: trust in aesthetic surgeon and/or other medical staff in the process of medical aesthetic encounters. These results call for further studies outside of primary care and a deeper understanding of how these ‘voluntary’ medical specialties work and influence patients/clients and their ‘treatment’.

## Introduction

Trust is “*confidence in the reliability of a person or system, regarding a given set of outcomes or events, where that confidence expresses a faith in the probity or love of another, or in the correctness of abstract principles*” (Giddens, 1991, p. 34). Trust is a fundamental element in people’s decision-making processes. In general, people tend to trust people they know more than abstract systems (Meyer et al., 2008), which have been studied in sociology for several decades (Giddens, 1991; Luhmann, 1979). In contemporary society, a certain level of trust in medicine, technologies, and sciences is necessary for people and populations to access the healthcare they need for their health (Solomon, 2021), even though they are not familiar with these systems and do not know precisely how they work.

Especially in medicine, trust is a source of sustainable relations between patients/clients^1^ and doctors (aesthetic surgeons). It influences a woman’s decision to undergo treatment that, in the context of this article, is an aesthetic/cosmetic surgery procedure(s). Aesthetic (or cosmetic)^1^ surgery is “*surgical procedures that alter, change or modify the surface, function, and appearance of the body purely for aesthetic purposes*” (Cook and Dwyer, 2017, p. 893). Existing sociological research on aesthetic/cosmetic surgery primarily focuses on women’s motivations that influence them to undergo aesthetic/cosmetic surgery procedures (Derakhshan et al., 2022), litigations (Macgregor, 1984), the reshaping/reconstructing of race/ethnicity, gender, and age (Arian et al., 2023; Menon, 2016), and how social media and other cultural factors can influence why women pursue aesthetic/cosmetic surgery (Arab et al., 2019; Furnham and Levitas, 2012). Collectively, this research has suggested that there exist social norms in which women’s bodies need to be altered to become “acceptable,” including aligning to what is considered to be “beautiful” and “normal.”

Historically, in sociological and feminist research, there have been two perspectives regarding women’s uptake of aesthetic/cosmetic surgery, as comprehensively examined by Cook and Dwyer (2017). One perspective suggests that aesthetic/cosmetic surgery is a form of gendered oppression that involves patriarchal control over women and women’s bodies. In reflection of Western society’s unrealistic and ideal feminine beauty norms, aesthetic/cosmetic surgery is a means to correct physical “defects” that deviate from these ideals of beauty and youth. In this process, women are victims of structural patriarchal control and manipulation. The other perspective, which Cook and Dwyer (2009) label as post-feminist arguments, suggests that women are aware of these beauty standards but are not tricked by them. Rather, aesthetic/cosmetic surgery can allow women to assert agency, control their bodies, and experience liberation. This is not to achieve the dominant feminine ideal but rather to look “normal” (Cook and Dwyer, 2017). As such, by aligning with social norms and expectations, women can achieve improved self-esteem, self-confidence, and self-perception (Al Ghadeer et al., 2021; Kazeminia et al., 2023). Of note for our research, these outcomes or possibilities are not achievable without trust being developed across the different levels including before and during the aesthetic encounter.

Despite the wealth of existing social and feminist research on women and aesthetic/cosmetic surgery, there is a lack of sociological problematization of what happens between women (potential clients/patients) and aesthetic surgeons during the consultation process at the clinic of aesthetic/cosmetic surgery. In this article, we focus on how trust is created between aesthetic surgeons and women patients/clients and the role of trust in decision-making processes that lead to undergoing aesthetic surgery procedure(s). During the medical encounter, women may seek to develop feelings of trust with medical professionals that can influence their decision to proceed—or not—with aesthetic/cosmetic surgery. While trust is commonly acknowledged as necessary in professional interactions, it is nonetheless a complex process that needs to involve two or more parties. This article explores the important individual factors and strategies for trust-building and the role of trust in decision-making between potential patients/clients, aesthetic surgeons, and/or medical system.

## Background

Decision-making is about choosing. By repeatedly making choices, social reality is continuously constructed. Sociological research on decision-making focuses mainly on how social structures and institutions construct, constrain, and control individual choices. According to Tallman and Gray (1990), individual decision-making processes or choices are constructed, constrained, and controlled through socialization and other historical, structural, and cultural forces. This includes considering the influential role of ideology, belief systems, wealth distribution, and technological change and progress (Sofo et al., 2013).

Decision-making processes also require developing and maintaining trust (Güroğlu et al., 2009). According to Simmel (1950), trust is conceptualized as enabling social action in decision-making situations where the actor is not entirely sure about the course of future events. Therefore, the primary function of trust is to facilitate negotiation between individuals (Luhmann, 1979). According to Giddens (1991: pp. 38–39), trust is essential to ontological security. It denotes an optimism that things will generally turn out “okay” in the end, as well as to create a sense of confidence in the world or what he refers to as the “existential anchoring of reality.” For example, he suggests that positive relationships early in life with caregivers produce basic trust in individuals, developing a sense of dependability in others and one’s surroundings. Significantly, trust is not only established between two persons but is also a link between the individual and wider social systems, including social institutions (Giddens, 1991).

In the social sciences, there is often an attempt to distinguish between intrapersonal (or facework) trust, which may characterize a specific doctor-patient relationship, and general (or faceless trust—institutional, social, or systemic trust)—which refers to attitudes toward social organizations (Hall et al., 2002; Luhmann, 1979). In this latter form of trust, which can be seen as nonpersonal, it is possible to further distinguish between a known institution, such as a specific medical clinic, and trust in the broader social or professional system (Hall et al., 2002), which can encompass medical knowledge. Social theorists have long argued that trust diffused within broader social and occupational systems is essential for the functioning of modern societies (Fukuyama, 1995; Luhmann, 1979). The stronger the trust at the systemic level, the easier it is for individuals to form interpersonal relationships without comprehensive knowledge of individual and personal characteristics. This type of trust is significant in modern societies because people must have a certain level of trust in, for example, science and technology, even though they are not entirely familiar with these areas (Solomon, 2021).

The healthcare system is an important social institution where the development of trust is vital for individual and collective acceptance of its dominance over health and well-being. This includes accepting medical specialists as sources of medical authority and knowledge (Williams and Calnan, 1996). As such, trust is a key component of the relationship between physicians and patients.

There is an extensive history of research on doctor-patient relationships and communication in primary care [see, for example, Arber (2008); Flatt et al, 2013; Mattson and Roberts (2001)]. Scholars interested in the healthcare encounter have long recognized that medicine is communication-intensive (Thompson et al., 2003) and that physicians’ conversational techniques have consequences that affect patients’ satisfaction with care, their adherence to recommended treatments, health outcomes, and litigation risk (Brown et al., 2003; Stewart and Ryan, 2003). The decision to opt-in for services is based on the patient’s expectations of interactions with the physician (Roter and Hall, 2006). Trust is crucial in the relationship between the service provider and the receiver (patient/client), serving an important function by creating intrinsic value in the medical relationship (McDonald and Heydenrych, 2022). Building interpersonal trust is considered crucial for achieving better therapeutic results, increasing patient happiness, and ensuring the patient’s medical ‘compliance’ (Van Den Assem and Dulewicz, 2015). Patients place equal value on a clinician’s interpersonal abilities, including building a trusting relationship with them, as they do on the clinician’s technical expertise and knowledge (Hall et al., 2002). Physicians must be able to gain the trust of new potential patients who, previous to the medical encounter, may know nothing about the physician. This process depends on the patient’s general perceptions and beliefs about the physician and the health care system in general (Axelrod and Goold, 2000; Parsons, 1951).

While there has been a sociological examination of the patient-doctor relationship and trust-building process, most studies have been done in primary or acute care systems, where deferral is made to doctors’ decisions and their expertise (Chipidza et al., 2015). Some research has also examined specialized settings such as pediatric encounters (Stivers, 2002), oncology (Beach et al., 2005), and general physicians (also called local doctors or primary care doctors) (Boubshait et al., 2022). Patients depend on the doctor’s knowledge, abilities, and goodwill in these cases. These cases have an inherent power imbalance between the patient and physician, and trust becomes essential to the treatment process. In this article, our concerns are not about how trust is developed in the medical approach to and treatment of serious or life-threatening illnesses but how trust is developed during aesthetic/cosmetic surgery decision-making, something in which treatment is not necessary or required. This development of trust outside of primary and acute care settings has been overlooked in sociological research. Yet the field of aesthetic/cosmetic surgery is different and unique because it encompasses both the medical field and an elective relationship that makes it possible to perform procedures on healthy (in contrast to unhealthy) individuals (McDonald and Heydenrych, 2022). Furthermore, little sociological attention has been paid to what occurs between clients/patients and aesthetic surgeons during their encounters. This has occurred despite the blurred borders of power and roles (client vs. patient, doctor vs. helper vs. businessperson, etc.) that exist in these encounters (Honelova, Vidovićova; in review). These gaps are addressed in our article.

Despite the aesthetic surgeon’s prominent role in the decision to undergo aesthetic/cosmetic surgery, this specialty is more than other medical disciplines marked by commercialization, with the paying client-provider relationship disrupting the decision-making asymmetry between patient and doctor. Potential patients/clients come to the clinic as healthy individuals with specific demands and expectations about the surgery that they are proposing (for example, breast augmentation). On the one hand, some aesthetic surgeons use the legitimacy of sovereign medicine (Novotný and Svobodová, 2014), which supports their expertise and work. On the other hand, women-clients/patients may come to the clinic with knowledge of the body modification or intervention that they desire and expect (or demand) a specific service from the aesthetic surgeon. That is, women have certain expectations from the service (aesthetic procedure) they choose (Mirivel, 2007) and assume that the aesthetic surgeon will meet their expectations both in terms of the “technique” of the procedure and the aesthetic outcome. This is because aesthetic/cosmetic surgery is done purely for aesthetic reasons that are not medically necessary and are based primarily on (individual) aesthetic concerns (Griffiths and Mullock, 2018). This contrasts with the traditional diagnostic approach of medicine when physicians diagnose patients with a disease or illness and subsequently lead and prescribe a treatment for patients who may have life-threatening conditions.

There is limited research on the role of aesthetic surgeons in patient/client decision-making and trust-building processes. Existing studies emphasize that aesthetic surgeons use both a personal, subtly erotic approach (for example, Spitzak, 1988) and technical means, such as before and after photographs or visualization (Blum, 2003), to induce hesitant patients/clients on the expertise of the provider. This advertising of the aesthetic surgeon’s work and skills is done to assure the patient/client of their skills and to undergo the procedure. Expertise based on previous work and friendly/erotic communication aims to evoke certainty in women and create trust in the aesthetic surgeon. Patients/clients are asked to present their problems or treatment plans/preferences in consultation with an aesthetic surgeon who then evaluates the individual’s requests and whether they are ‘reasonable’ (Hostiuc et al., 2022). The patient’s/client’s preferences are a significant determinant of the treatment chosen. Generally, aesthetic surgeons and patients/clients have the same goal, but each side approaches the “problem” from a different angle and perspective. However, how aesthetic surgeons conceptualize, manage, and negotiate their power as medical experts when interacting with their potential patients/clients remains unexamined.

## Methods

The data presented in this article represent a subset of a more extensive project titled Anti-ageing aesthetic surgery as a social construction of (non-) ageing and old age of women: The Phenomena of aesthetic surgery in the Czechia. The main aim of the project is perception of beauty and ageing toward cosmetic/aesthetic surgery and the journey leading to the cosmetic/aesthetic surgery.

In the first phase of the project, the entry criteria for participants were middle-aged women (aged 30–55 years) who are Czech nationals and have undergone or are planning to undergo anti-aging aesthetic/cosmetic surgery procedures. Study participants were recruited through posts on social platforms as well as the snowball method, which involved spreading word-of-mouth about the project. In all cases, potential participants were provided with full information about the project before they consented to participate. This process resulted in recruiting fifteen women (Table 1), with whom in-depth semi-structured interviews were conducted by Author 1 between December 2022 to May 2023. All interviews were conducted in locations according to the participant’s preferences to ensure a safe and comfortable place, mainly cafes or the participants’ homes.

**Table 1:** Information about aesthetic surgeons.

	1	2	3	4	5	6	7	8	9	10	11	12	13	14	15	16	17	18
Name	Kate	Lena	Barbara	Victor	Igor	Adam	Marcel	George	Adele	Pavel	Samuel	David	Susan	Michelle	Charles	Marika	Jane	Sylvia
Age	39	59	53	44	46	44	79	46	47	66	57	64	49	64	44	47	32	32
Type of practice	Owner	Employee	Owner	Owner	Head of the clinic	Owner	Owner	Head of the clinic	Owner	Head of the clinic	Owner	Owner	Owner	Head of the clinic	Head of the clinic	Surgery nurse	Manager of clinic	Manager of clinic

In the second phase, Author 1 conducted eighteen in-depth interviews with individuals working in aesthetic/cosmetic surgery clinics (Table 2). This included aesthetic surgeons, an operating nurse (one interview), and managers of aesthetic/cosmetic surgery clinics (two interviews). These interviews were guided by an interview guide and took place between May to December 2023. The criteria for research participation in the second phase included working in an anti-aging aesthetic/cosmetic surgery and the place of practice, which was restricted to the three largest cities in the Czech Republic (Brno, Prague, and Ostrava) where most aesthetic/cosmetic surgery clinics are located (McLaren and Kuh, 2004). We identified potential study participants via their practice website, from which we gathered data on location and the procedures offered to ensure the clinic met the study criteria. From this, we sent an informational email to 98 potential participants meeting the study criteria, in which we described the purpose of the research and opportunities for participation.

**Table 2:** Information about female participants.

Pseudonym	Victoria	Valery	Emily	Michelle	Olivia	Jane	Caroline	Lucy	Rosarie	Casandra	Ariel	Rebeca	Stella	Samantha	Erica
Age	40	43	33	52	39	32	49	42	38	31	35	55	36	42	31
Education	University (Master)	University (Master)	University (PhD)	University (Bachelor)	High school	High school	High school	High school	High school	University (Bachelor)	High school	High school	University (Master)	High school	University (Master)
Marital status	Married	Married	Single	Divorced	Partner	Married	Divorced	Single	Married	Single	Partner	Married	Partner	Married	Engaged
Children	2	0	0	1	1	0	2	1	0	0	0	2	0	1	0
Job	Project manager	CEO	University teacher	Policewoman	Assitant of ceo	Nanny	Director in kindergarten	Real estate	Businesswoman	Accounter	Logistic	Nanny	Administrative poistioon	CEO	People manager

In both two phases of the project, the interview questions focused on the patient/client encounters, including communication, negotiation, expectation(s), trust, and the decision-making processes. As Author 1 moved through the interviewing process, they frequently adapted the interview guide to reflect new lines of inquiry. As part of this process, Author 1 acknowledged the sensitivity of this topic by modifying the interview structure to reflect the participants’ specific contexts and their responses to the interview questions. Interviews lasted between 60 and 90 min. All interviews (except three with aesthetic surgeons) were recorded and transcribed with the participants’ informed consent. Three interviews that were not recorded were annotated by Author 1 during the interview, with these notes subsequently clarified and modified by the aesthetic surgeon participants.

In the third phase, interviews were also supplemented by non-participatory observations. This involved Author 1 spending time with women participants (the same as those who were interviewed) during different activities. One of the activities was attending appointments with aesthetic surgeons. In total, six visits between four participants and their aesthetic surgeons were attended by Author 1, with each appointment lasting between 15 and 30 min. Author 1 was introduced to the aesthetic surgeon as an accompaniment and researcher. During these appointments, observation notes were taken on selected elements of the medical (aesthetic) encounter, such as the communication process between the participant and the aesthetic surgeon, communication techniques (words used) by the aesthetic surgeon, the participant’s reactions and behavior during the medical (aesthetic) encounter, and Author 1’s discussion with participants about the consultation immediately after the encounter. These notes helped to provide a deeper understanding of the medical encounter, how women make decisions and act in different situations, and what meanings the women attach to particular actions and choices.

In the fourth phase, Author 1 also volunteered at one aesthetic surgery clinic. During this time, Author 1 helped at reception and attended six medical aesthetic encounters of between 15 to 30 min with random potential patients/clients. All employees, as well as patients/clients, were informed about the research position of Author 1, and everyone involved gave informed consent about Author 1’s presence during the encounter. During these appointments, Author 1 made observation notes on the same topics noted in the previous paragraph.

We acknowledge that Author 1’s presence in both cases could influence the natural process of medical aesthetic encounters and the behavior of aesthetic surgeons and patients/clients. We reflect on this potential bias in our analysis. This article draws on interview and (non)participatory observation data. When presenting the data, we clarify whether this is interview or observation data (Fieldnote).

All interviews were recorded and then transcribed verbatim with the participant’s consent. All interviews were anonymized, and pseudonyms were based on random calendar names. The interview transcripts and observation notes were uploaded to Atlas.ti for coding, and the transcripts were analyzed by the author team. We followed the principles of thematic analysis (Braun and Clarke, 2006; Ezzy, 2002) to identify key features and rhetorical tools participants employed to describe their decision-making and trust-building. Our analysis mainly focused on the part of the interviews when the participants were asked how they associate and describe trust and the decision-making process. Authors focused on entire narratives/stories contexts to capture and map different personal conceptions of how women and aesthetic surgeons understand and perceive the decision-making process and building trust and what those concepts mean to them. Detailed field notes ensured that the depth of context can persist with the data to allowed us to conduct robust research in line with qualitative approaches (Phillippi and Lauderdale, 2018).

The research received ethics approval from the Committee for Ethical Research of the Charles University.

## Findings

Through our study, we found that, in general, women’s decision-making and trust-building were developed in, shaped by, and revisited across a series of medical (clinical consultations) and non-medical encounters (friends, family relatives) as well as studying the available information (non-medical expertise). The decision-making of aesthetic surgeons was generally based on consultation with the (potential) patient/client, such as assessing the woman’s needs and the subsequent decision on whether and to what extent the cosmetic procedure was needed. The decision-making processes from both sides are based on ‘relational autonomy,’ where decision-making occurs in a co-production of interested dependence and within encounters with other people.

Based on our analysis, we developed three levels of trust building that influenced women’s and aesthetic surgeon’s decision-making processes on receiving or providing aesthetic/cosmetic surgery: (1) macro level: trust in medicine as a social institution and the specific aesthetic surgery clinic; (2) meso level: trust in other medical staff and aesthetic surgeon developed prior the medical aesthetic encounter; and (3) micro level: trust in a specific aesthetic surgeon and other medical staff, built while visiting the aesthetic surgery clinic. Categories can be linked, but we use them as an analytical tool.

### Macro level: trust in medicine

At the most general level, women had trust in medicine as a social institution. Women believed that the Czechia medical system worked well and that they could trust it. This trust emerged from understanding medical information communicated by their doctor/s and their previous interactions with other physicians. In their interviews, many women stated that they would not have aesthetic/cosmetic surgery anywhere abroad because they have less confidence in that medical system compared to the Czechia one, which they believe has higher quality standards, including the expertise of physicians:

“[I] *certainly have more confidence in Czech medicine and medical system.*” (Mrs. Rosarie, age 38).

In this case, participants shared their experience living abroad and with other medical systems. They were able to compare two medical systems, believing that the Czech medical system is of higher quality and that Czechia is more trustworthy for her than the other countries. In familiarity, past experiences are condensed, and their continuity is assumed, allowing for future-oriented trust. Their past positive experiences with the Czech medical system allow participants to believe that the Czech medical system is trustworthy, which influences their expectations of future experiences. In other words, individuals tend to trust things they are familiar with.

Some women also articulated this general trust as a trust in a specific aesthetic/cosmetic surgery clinic as a representant of the medical social institution:

“*So, I went there because I actually had confidence in the institution. And… I’ve never actually heard anything bad about* [the clinic].” (Mrs. Samantha, age 42).

This general trust was based on the clinic’s generally positive feedback, including word-of-mouth, name, brand, and reputation, as shaped by the history, surgical outcomes, and feedback on the clinic in the (online) media. In addition, some women believed that if the prices of the procedures were high, it was a guarantee of quality they could trust:

*“I’m still of the mindset that higher price equals higher quality, so I would probably look for clinics where the price is maybe, like exorbitant. And I would feel like I could find that quality*.” (Miss Casandra, age 31).

Conversely, women with higher socio-economic status tended to opt for smaller “no-name” clinics that offered greater intimacy and privacy. For them, these aspects created a sense of trust because undergoing aesthetic/aesthetic surgery was a personal and intimate experience for them:

*“I’m not really a believer… I do not believe in marketing. So when it’s the most well-known ones* [aesthetic surgery clinic] *that you see everywhere, and it does not seem they know each other here* [aesthetic surgeons]*, and you learn a lot* [gossip about other clients/patients and aesthetic surgeons]*. So, I try to stay away from those* [aesthetic surgery clinics]” (Mrs. Victoria, age 40).

Participants noted that gossip about clients/patients is an abuse of trust, and the need to rely on confidentiality with the aesthetic surgeon and/or aesthetic surgery clinic. Participants were convinced that almost everyone knew each other in these medical circles and a visit to a reputable, well-known aesthetic/cosmetic surgery clinic could be akin to a visit to, as noted by Mrs. Rebecca (age 55), “*a live television broadcast where women would publicly admit their body modification*.” In other words, gossip is often present in these clinics and among their patients/clients.

Because women such as Mrs. Victoria try to keep their visits to aesthetic/cosmetic surgery clinics secret, avoiding large aesthetic surgery clinics was vital to preserve her privacy. These concerns expanded beyond the clinic itself and included considering the clinic location and car parking availability:

“*I still choose* [the aesthetic surgery clinic] *based on parking - it’s an important thing. Because you do not want to be seen ….*” (Mrs. Victoria, age 40).

In this case, the general trust in medicine or specific aesthetic surgery clinics emerges from the confidentiality they can offer. This confidentiality is not only a perception of the patient/client but a marketing tool of the clinics, where some clinics advertise on their websites discretion in the clinic location and car parking as a guarantee of trust or “why” women should choose them.

Aesthetic surgeons also emphasized the importance of creating trust with potential clients/patients through their medical knowledge and experience, as well as staff professionalism across their entire aesthetic/cosmetic surgery clinic. Aesthetic surgeons believed they should present their expertise and skills through their work (for example, photographs of before and after aesthetic/cosmetic surgery). According to some aesthetic surgeons, examples of their work, including positive post-surgery outcomes, can create a trusting environment for potential patients/clients and can encourage women undergoing aesthetic/cosmetic surgery and support these women in deciding that their clinic is a good decision as well as undergoing cosmetic/aesthetic surgery in general.

### Meso level: *a priori* trust in the aesthetic surgeon

This category represents how trust is developed in aesthetic surgeons and other medical staff prior to the medical encounter. In these cases, the decision-making process was based on and influenced by pre-encounter trust. For example, some women trust aesthetic surgeons because they are doctors. Therefore, their medical qualifications, recognition as qualified doctors, and acceptance to practice in the Czech medical system allowed women to develop trust prior to the medical aesthetic encounter.

“*So, I’m like… I’m probably - maybe naive, but just like the doctor, I a priori trusted him.!* ……*And so, I went in, I’d already sort of decided, and I chose him with the confidence that he was the best then; everyone says so, so he must be, and also, the clinic had a great reputation*.” (Mrs. Rebecca, age 55).

For Mrs. Rosarie and other participants similar to her, the expert power and knowledge of the aesthetic surgeon were unquestionable. In this way, their qualifications and acceptance as medical professionals translated into trusting their professionalism and the surgical outcomes that they could achieve. Trust in the aesthetic surgeon was therefore reinforced by trust in the institution; for patients/clients such as Mrs. Rosarie, trust is not built through the patient/client’s interactions with the individual surgeon or the clinic. Rather, trust in the aesthetic surgeon is built through networks and/or on the general perception of the medical system and is supported by positive feedback gathered through social media and other medical environments or encounters.

Also, even though aesthetic/cosmetic surgery procedures are an elective medical service that women pay for, women may believe aesthetic surgeons to be prestigious people who are very busy and capable and should be afforded respect and esteem. This means that women may question their uncertainty and information-seeking and believe they should defer to medical expertise rather than raise “what if” questions. This perception reinforces and perpetuates the medical expertise and privilege of the aesthetic surgeons over women’s choices on their bodies.

Some women noted how the presence and interaction of the aesthetic surgeon with other medical team members (for example, nurses and general staff) and the functioning of the clinic in general also played a role in fostering trust.

“*So I went to the clinic, and when I was admitted there, one of the nurses and I started talking. She told me that if she had a choice, she would also go to Dr.* [the name of the aesthetic surgeon]*. And I would say that was the clincher for me. I had no argument when the nurse there knew all these doctors and told you this.”* (Mrs. Victoria, age 40).

In this case, participants developed trust in the aesthetic surgeon and the clinic based on a friendly interaction with one of the nursing staff. This allowed her to develop trust in the staff employed in this aesthetic/cosmetic surgery clinic, which also reflects their trust in the medical institution more broadly.

This importance of staff in developing trust in potential clients/patients and in recruiting the right staff who reflect the values of the clinic was noted by aesthetic surgeons:

“*Anyway, it’s all about the staff. If you do not have good people with a heart for the job, thinking about it, you probably cannot do anything with the best laser in the world. So, I would say that the most important thing is the human factor*.” (Dr. Adele, F, aesthetic surgeon, age 47).

Aesthetic surgeons are aware that women go to the clinic to spend money, so the whole experience has to be to the potential client/patient’s satisfaction and turn, impact their potential client/patient’s trust-building in the clinic and staff. This was described by the manager of the aesthetic surgery clinic, Jane, who noted, as part of the trust-building process *“aesthetic clinics must differentiate themselves in appearance from medical clinics”* (Miss Jane, F, the aesthetic surgery clinic manager, age 33). This perception of aesthetic clinics is based on the participant’s belief, that how a space looks and feels must encourage potential patients/clients to spend money and, therefore, must be similar to non-medical consumer spaces such as beauty salons:

“*Aesthetic surgery clinics try to make the space not look completely medical, but more… like more spa… It’s luxurious just* [the space of an aesthetic surgery clinic], *so it’s just, it feels like, a place where I understand I’m going to spend money.*” (Miss Jane, F, the aesthetic surgery clinic manager, age 33).

On the other hand, other participants preferred strictly medical visage of the clinics and believed, the trustfulness is based on medical authority which was represented by the space and dress codes of the (non-)medical staff.

### Micro level: trust in the aesthetic surgeon and other medical staff

This category represents how trust is built between the potential patient/client and the aesthetic surgeon during the aesthetic encounter. At this level, aesthetic surgeons play an active role through their actions, behavior, and work that (inevitably) influence women’s decisions and trust formation. For aesthetic surgery to occur, the aesthetic surgeon must gain the trust of potential patients/clients who know nothing or very little about them (beyond the information on the websites of the clinics, internet or concretely on the social media). In aesthetic surgeons’ words, “*trust comes first*” (Dr. Igor, M, aesthetic surgeon, age 46).

For potential patients/clients, the communications from the aesthetic surgeon were described as practical in obtaining information, negotiating the terms of surgery, and establishing whether surgery was necessary. This type of interaction established their expertise and, through this, helped to generate feelings of trust:

“*Medical perspective. I mean, it seemed to me that, like… I was like, well, this one probably understands it, so maybe she knows what she’s doing*” (Miss. Erica, age 31).

The trust in the medical expertise of a specific aesthetic surgeon was strengthened when women had previous aesthetic surgery and were pleased by the results. This is similar to the macro-level of trust building, though here, this is about individual relationships rather than a relationship with an institution. This is represented by Mrs. Valery:

“*Absolutely, because I am always satisfied with the result and know that he is really an expert in his field… If I could not trust him, I probably would not be able to be with him and put myself in his hands….”* (Mrs. Valery, age 43).

From the observations and interviews, it was prevalent that personal interaction with the aesthetic surgeon was a key component of women’s trust-building. Some aesthetic surgeons used a “friendly” and sometimes slightly “erotic” approach to encourage open discussions during the aesthetic encounter:

“The aesthetic surgeon explained the aesthetic/cosmetic surgery process in a friendly voice, still smiled at the participant, and asked if she understood everything. Also, he used the phrase, “*You are a beautiful woman. Are you sure you need the surgery*?” The participant seemed to be flattered and smiled, too.” (Field notes, October 22nd, 2023).

This friendly or erotic approach was often complemented by a technical and expert approach through the use of medical jargon and terminology.

Some aesthetic surgeons also believe that trust-building can involve saying “*No*” to women’s requests for specific forms or types of aesthetic/cosmetic surgery. This was explained by aesthetic surgeon Dr. David:

“*You have to be able to say no. Those women will come back to you* [for another procedure] *when they understand that you meant well by them…of course, I have an economic incentive to get that person to have surgery from me. But…I’m far more economically incentivized to have happy patients. Which sometimes means not operating*.” (Dr. David, M, aesthetic surgeon, age 64).

In this case the aesthetic surgeon’s ability to say “no” to surgical requests from women means they can trust that surgery will not be done at any cost and that they can invest their trust in his medical expertise to make the best decision. For Dr. David, refusing to perform specific aesthetic procedures is part of the trust-building process. It can result in women coming back with “other problems,” demonstrating to Dr. David that they trust his decisions and points of view as medical experts.

Although trust in medical knowledge/expertise and aesthetic surgeons was an important point in women’s decisions to undergo an aesthetic procedure, in some cases, the aesthetic surgeon’s enforcement of their medical expertise translated to a perceived paternalistic approach that was „detrimental” to developing trust with their potential patient/client:

“*I remember one* [aesthetic surgeon]. *Well, he was a very arrogant man. He hardly let me speak at all. He refuted everything I wanted and forced his procedures on me, which I did not want. He almost told me I should be quiet if I did not understand it and that he was the expert, yet he knew best how to do it. Well, I left the clinic quite horrified, wondering if this is how things are supposed to work normally, that I do not like this approach at all*.” (Mrs. Valery, age 43).

In cases such as Mrs. Valerie, women were unable to establish trust in the doctor and chose not to undergo the procedure with them.

Gender influenced this assessment, with the physical appearance of an aesthetic surgeon who was a man being less important than that of an aesthetic surgeon who was a woman. However, for some women, the appearance of aesthetic surgeons who are men were described as Hollywood stars:

“*He* [aesthetic surgeon] *was so Hollywood. It’s a good thing I had freshly washed my hair because otherwise, I would have felt completely… standing there like he was over me. So, it was almost… how do I say …. intimidating.”* (Mrs. Rosarie, age 38).

In this encounter, participants are not only evaluating the appearance of the surgeon, but they are also evaluating their own appearance in relation to him. Therefore, the “intimidating” performance of the aesthetic surgeon helped to reinforce their own feelings that they needed aesthetic surgery. At the same time, the charm of aesthetic surgeons was a factor in developing trust.

The appearance of an aesthetic surgeon who is a woman played a more significant role for some women in their decision-making process and trust-building, as opposed to how aesthetic surgeons who are men were discussed.

*“My doctor, she actually looks like what I would imagine. So, actually, that’s where the trust is. For me now, it’s even higher thanks to this… Plus, she has the same personality as me when we talk. “*(Mrs. Stella, age 36).

In this case, the visage of the aesthetic surgeon guided trust-building and was reinforced by the personality of the aesthetic surgeon. Some women preferred it if the aesthetic surgeons had the same sense of humor, interests, and values. Notably, the appearance of women aesthetic surgeons is no guide to their medical knowledge or expertise, nor their skill in surgical procedures, but nevertheless played a role in trust-building.

During their interviews, women extensively explored their feelings and emotions toward aesthetic surgeons and the environment of the clinic in general (layout and modern look). Participants trusted aesthetic surgeons whom they felt they could connect with, including experiencing understanding, sympathy, and empathy from the aesthetic surgeon. Some participants also reported qualities such as kindness, friendliness, commitment to quality, being listened to, not being rushed, concern for the patient/client, and support contributed to trust:

“*Just that humanity and a very positive attitude. When I came for the consultation, the doctor smiled, asking me what was bothering me and how I would like to solve it. He then informed me what he could do, how he could do it, what it would entail, and how it could specifically work for me*” (Mrs. Valery, age 43).

Women also considered the importance of establishing trust through aesthetic surgeons’ understanding of what they require and meeting their demands from the procedure.

“*… They* [aesthetic surgeons] *should probably take their time with me. I should not feel I’m under pressure. It’s just a treadmill, that’s for sure. They should be able to answer all my questions and try to come out of the woodwork.*” (Mrs. Lucy, age 43).

Aesthetic surgeons also considered the importance of an emotional ‘bond’ with a potential patient/client and believed it is essential to trust building:

*“Um, I guess it’s hard to explain in words. It’s more of an in-between, more of an in-between on some sort of emotional side, I guess, where some vibes, some really like emotions. And that’s like, in the 17 years I’ve been doing aesthetics, I’ve already got that like in me. Like somehow cultivated*.” (Dr. Susan, F, aesthetic surgeon, age 44).

Although Dr. Susan speaks of the emotional side and feelings, the statement also evokes expertise created through experience and practice in the field. In other words, aesthetic surgeons ‘exactly *know what women want/expect/need*’ (Dr. Susan, aesthetic surgeon, age 44). Nevertheless, aesthetic surgeons link the emotional or psychological aspects with their medical expertise, and this creates an intimate and comfortable environment for their patients/clients:

“*The psychological side is very important. It’s about trusting the surgeon, knowing whether she/he is an expert or not, what kind of practice she/he has, what the environment of the surgery is like.*” (Dr. Lena, F, aesthetic surgeon, age 59).

### Limitations of the study

We are aware that our study also has some limitations. The study is limited by its small sample size and sampling techniques. All our participants were from the three largest cities in the Czech Republic. An exclusively urban sample of communication partners could produce a specific bias regarding over-representing selected (socio-economic) characteristics. In our sample, most women were from the middle and higher social classes. On the other, the limitations could also be counteracted. The study focuses only on women because it is still an important issue, as women are primary consumers of cosmetic/aesthetic surgery clinics. Also, participants were primarily from middle and higher social classes as most of consumers as cosmetic/aesthetic surgery is not accessible or affordable for all as the procedures are not cheap.

In the future, this raises a question about whether and how the socio-economic status of women influences their trust and questioning of medical authority. In some cases, we found different strategies for choosing aesthetic surgeons, and the trust-building process of women were based on their socio-economic status. Some higher-class women choose smaller clinics offering a more secure and trustful environment. Therefore, it will be important to analyze the data further to determine the impact of socio-economic status on women’s trust-building and decision-making process.

## Discussion

In general, aesthetic surgeons work with their patients/clients to shape the profile of an ‘appropriate,’ ‘acceptable,’ or ‘normal’ feminine body, defined by socially acceptable ideals of gendered body image (Parker, 2009). While aesthetic surgeons seek to assert their professional status and expertise during the consultation(s), the patient/client expects a service they are willing to pay for (or have paid for). This complex and negotiated process creates a bond between the aesthetic surgeon, the medical staff, and the client/patient.

Most participants trusted in Czech medical system on the general level. This trust can emerge from familiarity and familiarization as the familiarity is a precondition for trust (Luhmann, 1979). This can be connected also with familiar faces, which is from the psychological perspective, powerful tool in creating trust. We instinctively believe a face or voice we are familiar with; therefore, some aesthetic surgery clinics reach celebrities as representativeness of their brand.

Notably, developing trust was not just about the medical expertise of the aesthetic surgeons and their technical skills or the friendliness and communication between staff and potential clients/patients. Some women based their feelings of trust on the aesthetic surgeons’ appearance, feelings, and emotions. However, our participants did not include or mention (dis)trust in technology and modern methods in aesthetic/cosmetic surgery. Some research was already interested in how modern technologies influence the client/patient’s trust (McDermott et al., 2020). In this study, most female participants voiced worries about the perceived safety and use of modern surgical technology.

Although we have identified three types of trust in this article, these are not the only types of trust that can occur in the patient/client and the aesthetic surgeon relationship. The woman’s trust in the cosmetic/aesthetic surgeon (does not) help to build her self-trust to stand up to society and its scrutiny and judgments. This can be seen in the participant’s testimonies which talked about choosing a clinic of aesthetic surgery based on parking and/or location. The anonymity is seen as trust in the clinic, through which participants believe that they will not be disappointed in society’s acceptance of her as she is trying to “*not be seen*” publicly. This may manifest the fear that women who visit clinics of aesthetic surgery may be judged or stigmatized by society. This claim can be supported by research by Bonell et al. (2021), which noted that women who are seeking cosmetic/aesthetic surgery procedures could be potentially subject to experience adverse psychosocial outcomes and unfavorably by society. Also, other variables can influence trust-building and then decision-making, such as the influence of three parts, especially if the woman’s partner is present during the encounter. Sometimes, the partner/husband comes to the aesthetic clinics with the woman and ‘dictates’ to the aesthetic surgeon what to do with the woman’s body and how he/she should do it. According to Morgan (1991), women undergoing cosmetic/aesthetic procedures are victims of beauty dictates manipulated by their partners and aesthetic surgeons. She called that a ‘false consciousness’ where women believe they are making a voluntary choice (free will) but are merely conforming to prevailing cultural (male) ideas about the female body. Therefore, future researchers can discuss between whose parties the trust is established - partner-cosmetic/aesthetic surgeon, cosmetic/aesthetic surgeon-woman, partner- cosmetic/aesthetic surgeon-woman.

Aesthetic/cosmetic surgery clinics are becoming new kinds of beauty salons, and a gradual domestication of medical procedures can be seen. As Cook and Dwyer (2017) claimed, Botox is already beyond normalization and has become domesticated as a routine in everyday life. Although the procedures our participants underwent are not performed as frequently as Botox injections, the number of invasive procedures is increasing, and the perception of aesthetic surgery clinics as a place of rest is increasingly causing the normalization and standardization of these procedures. According to our results and discussion, further analysis of aesthetic surgery is needed as aesthetic surgery brings both positive and negative changes to societies. This research examined only a group of women and aesthetic surgeons. Future research could thus explore whether trust in cosmetic/aesthetic surgery differs based on gender and whether and how trust-building and decision-making would differ for male cosmetic/aesthetic surgery patients/clients.

## Data Availability

The datasets presented in this article are not readily available because participants did not give consent to share data. Requests to access the datasets should be directed to michaelahonelova@seznam.cz.

## Footnotes

^1^We have used both terms throughout the text because our participants used them interchangeably, which is also reflected in the research literature that we discuss later in the article.
